# Effects of a Portuguese social–emotional learning program on the competencies of elementary school students

**DOI:** 10.3389/fpsyg.2023.1195746

**Published:** 2023-05-16

**Authors:** Vera Coelho, Carla Peixoto, Helena Azevedo, Francisco Machado, Mónica Soares, Andreia Espain

**Affiliations:** ^1^University of Maia, Department of Social and Behavioral Sciences, Research Unit in Psychology and Human Development, Maia, Portugal; ^2^Center for Psychology at the University of Porto, Porto, Portugal; ^3^Center for Research and Innovation in Education (inED), School of Education, Porto Polytechnic, Porto, Portugal; ^4^Higher School of Education of Paula Frassinetti, Porto, Portugal; ^5^Associação Mente de Principiante, Maia, Portugal

**Keywords:** socio emotional learning, universal intervention, elementary school, self-regulation, communication, classroom peer relationships

## Abstract

**Introduction:**

It is widely recognized that socio-emotional learning (SEL) interventions can contribute to supporting students’ positive development of socio-emotional skills (SES) and positive relationships with peers and teachers. Thus, interest in promoting students’ SES through universal evidence-based programs is spreading around the world, including in Portuguese schools.

**Methods:**

This quasi-experimental study examines the efficacy of a SEL classroom-based program, infused into the curriculum, on students’ communication, self-regulation, and classroom peer relationships. Participants included 208 third- to fourth-grade students from three Portuguese public elementary schools: 143 in the intervention group (54.5% boys; *M*_age_ = 8.72; *SD* = 0.61); 65 in the comparison group (52.3% boys; *M*_age_ = 8.66; *SD* = 0.59). Measures included: Study on Social and Emotional Skills, parent, child, and teacher versions; and Classroom Peer Context Questionnaire, completed by students. The study followed a pre- and post-test design, with a 16-week intervention.

**Results:**

For the overall participants, results show a positive effect of the program on students’ assertiveness (family report), peer conflict and peer cooperation. Effects were analyzed separately by school grade. A statistically significant positive effect of the program on third-grade students’ assertiveness and sociability was found. For fourth-grade students, a positive effect was found on - emotional control). classroom conflicts, isolation, cooperation and cohesion behaviors.

**Discussion:**

These positive effects support the expansion of universal interventions when aiming at strengthening SEL in Portuguese school settings, underlining the relevance of embedding SEL into the curricula and daily practices at schools.

## 1. Introduction

Social–emotional learning (SEL) is an educational model aimed at improving students’ social–emotional skills (SES). SEL is usually defined as the process through which students develop a set of interrelated competencies that allow them to recognize and manage their emotions, set and achieve goals, and engage in responsible decision-making processes and positive interactions through the development, for instance, of perspective-taking, conflict management, and relationship skills [e.g., Collaborative for Academic, Social, and Emotional Learning (CASEL), 2021]. In the last decades, there has been an evident and growing interest in SEL, particularly in the field of educational psychology, as research shows that SEL fosters students’ SES, thus improving their ability to solve problems and engage in positive relationships with others and increasing their chances of success, both academically during their school years and throughout their adult lives (e.g., Weare and Nind, 2011; Pinto and Raimundo, 2016; Greenberg et al., 2017; Marchante and Coelho, 2021). Positive short- and long-term effects of SEL for students’ lives are underlined in the literature (e.g., Bradshaw et al., 2009; Durlak et al., 2011; Taylor et al., 2017). Therefore, in a constantly changing world, helping children and young people to develop the skills they need to thrive throughout their academic, professional, social, and personal lives becomes of paramount importance.

Schools are in a pivotal position to foster students’ SES, as children and adolescents spend a significant amount of their time in these environments and face several challenges, both academic and social, during schooling that require SES for positive development and learning [Greenberg et al., 2017; Nakano et al., 2019; Collaborative for Academic, Social, and Emotional Learning (CASEL), 2021]. Therefore, efforts to promote students’ SES through universal evidence-based SEL programs in schools are spreading around the world, including in Portuguese schools (Pinto and Raimundo, 2016; Bowles et al., 2017). The number of SEL programs has increased worldwide, as has the search for evidence of their efficacy. Nevertheless, the need to expand the research on developmentally appropriate SEL programs remains, particularly in Portugal, where several SEL programs have been developed and implemented in the last decades. In view of these considerations, the present study aims to analyze the effects of a SEL classroom-based program, infused into the curriculum, on the communication, self-regulation, and classroom peer relationships of elementary school aged children.

### 1.1. School-based universal social–emotional learning

Schools are considered a primary developmental context for children and adolescents, playing a central role in the promotion of students’ development of important life skills, including SES (Weissberg and Elias, 1993; Greenberg et al., 2017). Therefore, SEL has been described in the literature as a fundamental part of education (e.g., Durlak et al., 2011; Jones et al., 2015; Oberle et al., 2016). Research provides evidence that SES have an important impact on various developmental outcomes, including children’s school success and positive peer relations and emotional state (e.g., Jones et al., 2015; Greenberg et al., 2017; Ștefan et al., 2022), with lasting effects reported over time (e.g., Bradshaw et al., 2009; Denham et al., 2012; Taylor et al., 2017; Denham, 2018). Given the above, ensuring that SEL becomes an integral part of educational contexts, by including it in the school curriculum and culture (Domitrovich et al., 2010, 2017; Weare and Nind, 2011; Weissberg et al., 2015; Greenberg et al., 2017), is indispensable for achieving a healthy school climate. In this regard, schools are taking care to incorporate high-quality SEL interventions into their daily classroom practices, recognizing that academic skills and SES are interdependent and inseparable and should be developed jointly at school from an early age (FitzPatrick et al., 2014; Blewitt et al., 2020).

School-based universal SEL programs have been associated with positive outcomes for students (across all grade levels), such as the improvement of academic performance and SES and the reduction of stress levels and behavioral problems (Durlak et al., 2011). In their landmark systematic review, Durlak et al. (2011) underline that the development of SES contributes to better school adaptation and involvement, being associated with motivation for academic achievement. Moreover, when delivered effectively, SEL programs are associated with significant, and possibly long-lasting, benefits for different areas of students’ lives, including academic, personal, social, and professional areas. Studies show that the implementation of SEL programs at an early age is effective in fostering learning, a positive school climate, positive relationships, a positive self-concept, and increased well-being, as well as in decreasing behavior problems, drug use, and emotional distress (Durlak et al., 2011; Durlak, 2015; Taylor et al., 2017). Moreover, evidence from longitudinal studies indicates that such positive effects may persist for over 15 years on social, emotional, and behavioral outcomes (e.g., Taylor et al., 2017). Along the same lines, Greenberg et al. (2017) report that children with higher SES are more likely to succeed in their careers, develop positive relationships, have balanced mental health, and become engaged citizens later in life.

Nevertheless, there are contradictory findings (e.g., Zeidner et al., 2002; Carroll et al., 2020), with some studies reporting little to no evidence of effectiveness and recognizing that there is a need for greater efficiency in delivering universal SEL programs in schools without compromising implementation quality (Domitrovich et al., 2010). Consequently, the need to discuss both the quality of the intervention/program and the quality of its implementation has been emphasized (Durlak and DuPre, 2008). For instance, the quality of the implementation and dosage were identified as primary limitations of school-based SEL programs, associated with the lack of effectiveness of interventions (e.g., Embry and Biglan, 2008; Jones and Bouffard, 2012). Thus, a set of quality characteristics have been identified for universal SEL interventions, as well-designed and well-implemented school-based SEL programs are deemed the most likely to improve children’s outcomes (e.g., Durlak et al., 2011; Bierman and Motamedi, 2015; Taylor et al., 2017; Voith et al., 2020; Mahoney et al., 2021; Ștefan et al., 2022). Overall, it is recommended that SES are be promoted in safe and caring learning contexts by engaging teachers, other school team members, children, and families in SEL practices that build relationships in the school community and improve child competencies (Durlak et al., 2011).

In Portugal, in the past few years, the number of SEL programs in educational settings has also increased (Pinto and Raimundo, 2016; Bowles et al., 2017; Cristóvão et al., 2017; Peixoto and Coelho, 2022), particularly in elementary and middle schools (e.g., Raimundo et al., 2013; Coelho et al., 2016; Coelho and Sousa, 2017). An important contribution to this increase was the investment made by the Calouste Gulbenkian Foundation through the “Gulbenkian Academies for Knowledge.”^1^ Between 2018 and 2021, the Calouste Gulbenkian Foundation financially supported around 100 projects aiming to promote key SES in children and youth under 25 years of age, including the school-based universal intervention program “Calmly – Learning to Learn Yourself” [Calmamente—Aprendendo a Aprender-se]. Despite the increased investment on school-based SEL interventions, there seems to be a lack of knowledge of their effects on children attending Portuguese elementary schools, since experimental studies are scarce (e.g., Raimundo et al., 2013).

### 1.2. Social–emotional skills and social–emotional learning

Although there are a multitude of frameworks that address the field of socio-emotional skills, sometimes using different terminology to define and organize this research area (Taxonomy Project, n.d.; Berg et al., 2019; Djamnezhad et al., 2021), all frameworks include a large set of interrelated competencies [see Taxonomy Project, n.d.; OECD, 2019; Collaborative for Academic, Social, and Emotional Learning (CASEL), 2021]. Our study focused on self-regulation and communication skills due to their role in supporting relationships and children’s ability to manage their behaviors and emotions. Self-regulation skills in particular have been widely studied over the past years, with evidence supporting their associations with several child outcomes, such as learning, adjustment, engagement behaviors, and social competencies (e.g., Eisenberg et al., 2001; Olson et al., 2005; McClelland et al., 2007; Williford et al., 2013). Negative associations between self-regulation and later behavior problems have also been highlighted (e.g., Murray and Kochanska, 2002; Hughes and Ensor, 2011; Sawyer et al., 2015), with both emotional and behavioral regulation underlined as key aspects of self-regulation for children to adequately respond to academic and social demands in educational setting. Self-regulation skills have been constantly associated with decreased behavioral problems and increased engagement and prosocial behaviors across school years (e.g., Olson et al., 2005; Carlson and Wang, 2007; McClelland et al., 2007; Sawyer et al., 2015). For instance, research has demonstrated that students who participated in interventions focused on self-regulation show significant improvements in academic performance. In this scope, Durlak et al. (2011) found that students who participated in SEL programs focused on self-regulation showed significant improvements in academic achievement. Another study by Raver et al. (2011) found that kindergarten students who participated in an intervention that focused on self-regulation showed significant improvements in both academic achievement and behavior. Along the same lines, communication skills have also been widely studied, with the literature reinforcing that they are associated with important milestones of children’s socio-emotional development (e.g., Heberle et al., 2020; Rautakoski et al., 2021), being specifically identified as part of the core competencies for establishing and maintaining healthy and supportive relationships [e.g., Collaborative for Academic, Social, and Emotional Learning (CASEL), 2021]. Communication difficulties can negatively affect social interaction as well as emotional and self-regulation (e.g., St Clair et al., 2019), making it crucial for children’s success to develop a set of communication skills that allow them to function in different settings. Moreover, effective communication skills have been associated with better academic achievement and self-regulation outcomes (e.g., Ramsook et al., 2020). Hence, it is relevant that SEL interventions can foster children’s abilities to communicate clearly, listen, cooperate, and work collaboratively, which are key aspects for the learning process and consequently for academic achievement. Studies have investigated the relationship between SEL interventions and students’ self-regulation and communication. Studies found that students participating in SEL programs that focused on communication showed significant improvements in academic achievement and social competence (e.g., Jennings and Greenberg, 2009).

### 1.3. Classroom peer relationships and social–emotional learning

Schools, and particularly classrooms, are pivotal contexts for social interactions, challenging children to develop interactions and relationships among each other, promoting a positive classroom climate (e.g., Ladd, 2005; Denham et al., 2012; Boor et al., 2016). The literature on school climate and SEL highlights the role of relationships in school success and sense of well-being and quality of life (Thapa et al., 2013). It is recognized that SEL interventions serve as a way of fostering positive relationships with peers, teachers, school staff, and families, contributing for students’ ability to establish and maintain healthy relationships through effective communication, social engagement, and more collaborative teamwork [Collaborative for Academic, Social, and Emotional Learning (CASEL), 2017].

Peer relationships in classroom context are described as a relevant dimension for processual quality of classroom contexts (e.g., Luckner and Pianta, 2011; Rivers et al., 2013; Madill et al., 2014), which means that high quality relationships between peers in classrooms tend to promote children’s academic success and well-being (e.g., Androutsou and Anastasiou, 2014; Maxwell et al., 2017; Konold et al., 2018). These relationships are described as a complex phenomenon, with some authors proposing that to fully understand them we need to consider different levels of analysis, namely: individual level (e.g., characteristics children bring to social interaction, such as their social orientation to peers, social skills, and knowledge), interactional level (e.g., children’s dyadic day-to-day interactions and behaviors), relational level (meanings, expectations, and emotions that children have and express toward each other), and group level (e.g., patterns and characteristics of interactions and relationships present in a classroom, which reciprocally influence one another; e.g., Rubin et al., 2006; Boor et al., 2016). These levels of analysis are described as intertwined, which means that they are interdependent and should be viewed as a complex system. Additionally, literature describes that, simultaneously, not only children need SES (e.g., communication skills), to engage in positive peer relationships and interactions, but also interactions among peers themselves, also provide a fundamental context for the development of SES (e.g., Denham et al., 2012; Rivers et al., 2013).

Regarding the role of SEL in fostering classroom peer relationships, there is an assumption that SEL can function as means for children to acquire peer conflict resolution strategies, thus reducing impulsive behaviors (e.g., Bierman et al., 2016). Which implies that SEL can have an important role on reducing well-known issues that greatly interfere with school dynamics, social climate and effectiveness, namely aggressiveness and violent behavior, as impulsive behavior is a key aspect at the base of these disruptive behaviors. More broadly, research has also shown the negative impact of the lack of adequate peer relationships, underlining that peer relationship difficulties in childhood are predictors of future psychological maladjustment (Rivers et al., 2013; Sakyi et al., 2014; Shin et al., 2016). As SEL universal intervention in schools has been proven to foster improvements in children’s perception of warmth and connectedness with their peers, supporting the potential of SEL for enhancing classroom climate and promoting positive learning and development environments (Rivers et al., 2013), we can safely consider that SEL based intervention benefits not only present-day school contexts, but also student’s mental health and well-being going forward. The relevance and validity of supporting a wider implementation of SEL based intervention programs in school contexts has been continuously reinforced, as research keeps providing evidence of its effectiveness in terms of improving schools’ social climate and students’ mental health and well-being, as well as reducing the incidence of behavioral problems, namely violence. Over the years, studies have shown that school based SEL programs contribute to maintain stable, emotional, and supportive relationships, to promote significant changes in antisocial behavior, to a relevant increase in pro-social attitudes, as well as a decrease of students’ aggressive behaviors (e.g., Cooke et al., 2007; Zins et al., 2007; Durlak et al., 2011).

### 1.4. Study goals

Despite the increased investment of both practitioners and researchers in developing, implementing, and evaluating school-based SEL programs in the past years, the effectiveness of such programs remains unclear, particularly in Portugal, where few quasi-experimental studies have been conducted within this field (e.g., Raimundo et al., 2013; Voith et al., 2020). Considering this, the present study aims to analyze the effects of the school-based universal intervention program “Calmly – Learning to Learn Yourself” [Calmamente—Aprendendo a Aprender-se] on child SES, namely self-regulation and communication skills, as well as on peer classroom relationships.

Overall, self-regulation and communication are critical outcomes of SEL interventions once these can provide children with the tools they need to navigate the social and emotional challenges they face in and out of school. Moreover, literature has shown that peer classroom relationships are pivotal to promote a positive and inclusive classroom environment, improve academic outcomes, and develop essential social and collaborative skills that children need to succeed in school and across their lives. Regarding the self-regulation skills, the study focuses on two specific sub-dimensions, namely self-control and emotional control. Concerning communication skills, the study focuses on assertiveness, cooperation, and sociability specific subdimensions. Furthermore, and considering that research provides ample evidence that children participating in SEL-based programs tend to develop important skills for peer interaction, this study also explores potential effects of the SEL-based intervention program (“Calmly - Learning to Learn Yourself”) on children’s perception of their peer classroom relationships. More specifically, we aim to analyze if the program has positive effects on key variables of peer relationships, namely (i) the child level of comfort in peer relations in the classroom; (ii) the levels of cooperation and conflicts between peers in the classroom; (iii) the levels of mutual affection between peers in the classroom; and (iv) the levels of classroom group cohesion and isolation. Moreover, the study analyzes the effects of the program separately the whole group of elementary school, aged children participating, as well as for third and fourth grade students.

Building on previous research showing that SEL universal intervention can have a positive effect in several SES and classroom social climate variables, the following hypothesis were formulated: (i) the intervention program “Calmly - Learning to Learn Yourself” will lead to a statistically significant improvement in children’s self-regulation skills, specifically in the sub-dimensions of self-control and emotional control for third and fourth grade students, when comparing to children not attending the program; (ii) the intervention program “Calmly - Learning to Learn Yourself” will lead to a statistically significant improvement in children’s communication skills, specifically in the sub-dimensions of assertiveness, cooperation, and sociability, both for third and fourth grade students, when comparing to children not attending the program; (iii) children who participate in the intervention program “Calmly - Learning to Learn Yourself” will report a statistically significant increase in their level of comfort in peer relations, level of cooperation and group cohesion in the classroom, and levels of mutual affection between peers in the classroom, both for third and fourth grade students, when comparing to children not attending the program; (iv) children who participate in the intervention program “Calmly – Learning to Learn Yourself” will report statistically significant lower levels of conflicts between peers in the classroom and a decrease in isolation in classrooms, for third and fourth grade students, when comparing to children not attending the intervention program.

## 2. Methods

This study uses a quasi-experimental design, with a pre- and post-assessment and an intervention period of 16 weeks. An intervention group (IG) and a comparison group (CG) were included, with schools randomly assigned to each group. A multi-informant approach was employed, using self- and hetero-report measures.

### 2.1. Participants

The current study included 12 classrooms across three elementary schools in the North region of Portugal. Of these, six were third-grade classrooms and six were fourth-grade classrooms. Schools were randomly assigned to the IG (two schools, eight classrooms) and to the CG (one school, four classrooms). All lead teachers responsible for participating classrooms (*N* = 12) were included in the study, namely eight teachers in the IG and four teachers in the CG. Overall, the teachers were all female, with a mean age of 47 years (*M* = 47.18, *SD* = 8.2). The average number of years teaching was 23.36 years (*SD* = 8.64). All teachers had a higher education degree, with 16.7% teachers holding a master’s degree.

This study included 212 students (115 male) aged between 8 and 10 years (*M* = 8.69, *SD* = 0.61). From these, 145 children (79 male) were allocated to the IG and 67 (36 male) to the CG. In the IG, 63 students attended the third grade and 82 attended the fourth grade. In the CG, 32 students were in the third grade and 35 in the fourth grade. Independent *t*-tests revealed that there were no significant differences between students in the CG and those in the IG with regard to their age, *t*(210) = 0.67, *p* = 0.45. A chi-squared test for independence indicated that there were no significant differences between the CG and the IG in terms of gender, χ^2^(1) = 0.01, *p* = 0.92. Fathers from students in the IG were aged between 27 and 72 years (*M* = 41.72, *SD* = 6.86); the mothers’ ages ranged between 26 and 52 years (*M* = 40.23, *SD* = 5.41). In the CG, the fathers’ ages varied between 32 and 52 years (*M* = 41.37, *SD* = 4.28), and the mothers were aged between 30 and 53 years (*M* = 39.98, *SD* = 4.81).

### 2.2. Measures

The measures included both self-report questionnaires, completed by students, and hetero-report questionnaires, completed by teachers and families. All measures were completed before (pre-test) and after (post-test) the implementation of the intervention program.

#### 2.2.1. Study on social and emotional skills

The Study on Social and Emotional Skills (SSES; OECD, 2019) aims to assess the social and emotional skills of children. It is organized into six dimensions, namely self-regulation, communication, adaptability, creative thinking, resilience, and problem-solving. The SSES has one version for children, one version for families, and one version for teachers. All items are rated on a Likert scale ranging from 1 to 5 points (1—totally/completely disagree; 5—totally/completely agree). In this study, two of the six dimensions were used, namely self-regulation and communication. The self-regulation dimension includes 16 items organized into two subdimensions: self-control and emotional control. The communication dimension includes 24 items, organized into three subdimensions: assertiveness, cooperation, and sociability. In the present study, adequate values for internal consistency were found, with Cronbach’s alpha for all dimensions and subdimensions ranging between 0.66 and 0.83 for the child’s version, between 0.83 and 0.92 for the family’s version, and between 0.71 and 0.92 for the teacher’s version. For pos-test data, Cronbach’s alpha ranged between 0.84 and 0.87 for all dimensions and subdimensions of the child’s version; between 0.80 and 0.93 for the family’s version of the measure; and between 0.68 and 0.94 for the teacher’s version of the measures.

#### 2.2.2. Classroom peer context questionnaire

The Classroom Peer Context Questionnaire (CPCQ; Boor et al., 2016) measures the children’s perceptions of peer relationships in the class at the individual, interaction, group, and relationship levels (Fava, 2018; Hogekamp Fernandes, 2020). It includes 20 items, organized into six dimensions: Comfort in the classroom (individual level), Cooperation in the classroom (interaction level), Conflict in the classroom (interaction level), Mutual affection (relationship level), Cohesion of the class (group level), and Isolation (group level). Each dimension is composed of four items, except for Conflict (three items) and Mutual affection (one item). Items are rated on a 5-point Likert scale, from 1 (completely false) to 5 (completely true).

The CPCQ has shown good psychometric properties in its original study (e.g., Boor et al., 2016), as well as in previous studies conducted in Portugal (Fava, 2018). Internal consistency was analyzed in the present study through Cronbach’s alpha. Acceptable internal consistency values were found for all dimensions, except for Comfort. More specifically, Cohesion had a Cronbach’s alpha of 0.79 at pre-test and 0.78 at post-test; Conflict had a Cronbach’s alpha of 0.71 at pre-test and 0.67 at post-test; Comfort had a Cronbach’s alpha of 0.53 at pre-test and 0.71 at post-test; Isolation had a Cronbach’s alpha of 0.65 at pre-test and 0.64 at post-test; and Cooperation had a Cronbach’s alpha of 0.78 at pre-test and 0.83 at post-test. Considering the low internal consistency of the Comfort dimension at pre-test, the data on this dimension at this moment should be interpreted with caution.

#### 2.2.3. Sociodemographic questionnaires

Two sociodemographic questionnaires were developed. The student and family version of the sociodemographic questionnaire captures students’ and families’ sociodemographic characteristics (e.g., child gender, birth date, nationality, number of school retentions, the number of siblings, parents’ level of education, and employment status). The teacher version of the sociodemographic questionnaire was developed to gather sociodemographic data and information on the professional experience of the teachers, such as gender, age, marital status, number of years teaching, training completed, and employment status.

### 2.3. Procedures

Data collection followed all the ethical procedures according to APA standards. One school cluster was selected by convenience among the network of the researchers. The study was presented to the school director in a brief meeting. After getting the school cluster director approval for the study, a meeting was conducted with all elementary school teachers to present the project, study goals, and explain the randomization process. All teachers agreed to participate. Then, parents received a flyer presenting the study, as well as an informed consent form. A participation rate in the study of 79.4% of children in the study was achieved. Overall, written informed consent was obtained from the school director, teachers, and families. Data were collected at two time points—pre-test (December 2020) and post-test (June 2021)—for all participants in the IG and the CG. In accordance with ethical guidelines, participants in the CG had the opportunity to participate in a brief version of the intervention delivered to the IG after post test data collection.

Students completed the questionnaires in their school classroom in the presence of the researchers. The questionnaires for the families were sent in sealed envelopes, and families were asked to return them to their child’s teachers, also in sealed envelopes. Teachers completed the questionnaires individually. The same procedures were used both in pre- and post-test data collection, both to IG and CG. The intervention with the program “Calmly – Learning to Learn Yourself” [Calmamente–Aprendendo a Aprender-se] started in January 2021, after pre-test data collection, and lasted for 16 weeks. All students in the classroom participated in the intervention, as this was infused into the curricula, although not all students were included in the study due to lack of parental consent to be part of the study. The intervention was monitored through self-report measures completed by the facilitator, external observations, and regular supervision sessions. Pre- and post-test assessments were conducted by external researchers, with independent teams responsible for the external evaluation and for the intervention process. This procedure aimed to decrease the biases in the assessment procedures, particularly when completing the questionnaire with elementary school students.

### 2.4. Intervention: calmly—learning to learn yourself [calmamente—aprendendo a aprender-se]

“Calmly – Learning to Learn Yourself” [Calmamente—Aprendendo a Aprender-se] is a SEL-inspired universal program aiming to promote SES, with a mindfulness and growth mindset component. It is a classroom-based program infused into the school curriculum and aimed at facilitating the harmonious development of social and emotional skills, namely self-knowledge, adaptability, emotional regulation, communication, resilience, and problem-solving, among children and young people. It is supported by a set of dynamic teaching materials (e.g., card decks, personal notebook, and the “mini-calm cloud” (“mini-calma”)] designed to facilitate and enrich the intervention. Each session proposes the development of one or more socio-emotional competencies. The program is organized into 10 themes, such as Share, Breathe, and (being) Among Others, and it invites students to go a journey with several stops along the way, whenever a new theme emerges. Several strategies, such as posters, reflection/brainstorming, open questioning, modeling, social and self-reinforcing feedback, and group games, are used in the program sessions. Skills and concepts are typically presented through various challenges in each session.

The program is structured as a set of weekly dynamic sessions and was specifically developed to be implemented in school contexts. It is expected to be implemented throughout the entire school year. In the present study, 16 developmentally appropriate sessions of 60-min each, delivered weekly by a trained facilitator in the presence of elementary school teachers responsible for each classroom, were implemented. The program was organized in 16 sessions in order to ensure a weekly presence in the classrooms across the school days calendar between January and May, as well as to fit the schools availability for the curricula infusion. Under this program, the facilitator is expected to work in collaboration with the teachers, encouraging them to promote the generalization of the skills developed in the program during the week by expanding activities and reminding children to use the strategies learned during the day (e.g., doing breathing exercises when they feel anxious or replicating with their families the activities carried out in the sessions). The program facilitator follows the session plans available in the program manual. For each session, the manual provides information about the SEL objectives, the strategies to be implemented, and the materials to be used.

#### 2.4.1. Training, supervision, and monitoring

To implement the program “Calmly – Learning to Learn Yourself” [Calmamente—Aprendendo a Aprender-se], facilitators need to undergo certified training. The training program designed for the facilitators implementing the program with elementary school children within the scope of the “Gulbenkian Academies for Knowledge” encompasses 50 h, including both theoretical and practical sessions, with role-playing activities to train specific intervention competencies. The training was delivered by the program’s author. Throughout the whole intervention period, weekly supervision sessions lasting approximately 60 min were held individually with each facilitator to ensure program fidelity. Besides supervision sessions, facilitators also completed a questionnaire after each intervention session with the children in each classroom, making it possible to document the fidelity of the intervention, the dosage, and the children’s and teachers’ responsivity, and the implementation quality. The responsivity of the children, parents, and teachers was also captured through child, teacher, and parent satisfaction questionnaires, completed at the end of the intervention.

Regarding program dosage, the 16 sessions of the program designed were implemented, with four sessions being implemented online due to the COVID-19 pandemic. Adaptation of activities to the online format was designed in collaboration between the program facilitators and the program’s author. On average, children received 93% of sessions.

Implementation fidelity was self-reported by the facilitator of the intervention, for each session in each classroom. On a rating scale of five points, the facilitator registered the extent to which the session plan was accomplished according to the manual instructions and goals (1—not at all; 5—completely). Data from the face-to-face sessions (*n* = 12) revealed that on average, the sessions plans were almost completely accomplished (*M* = 4.63, *SD* = 0.21). Responsiveness was, on average good. For this indicator, the facilitator scored students engagement, level of positive affect and levels of satisfaction with each session. An average of the scores of the three items scored was computed for each session. Mean values were 4.60 (*SD* = 0.21), meaning that, in a five-point scale, facilitators perceive high level of students’ responsiveness to the intervention throughout the sessions. Levels of satisfaction were also collected (in a five-point scale), at the end of intervention. Satisfaction questionnaires were completed by students, parents, and teachers. Results showed that students were very satisfied with the program, with average satisfaction levels of 4.41 (*SD* = 0.67), in a maximum of 5. Parents satisfaction levels were in a medium-high level, with an average score of 4.10 (*SD* = 0.50). Teachers reported an average satisfaction with the program of 4.29 (*SD* = 0.48), with lower levels of satisfaction regarding the adequacy of the program length (*M* = 3.13, *SD* = 1.36), and with the program ability to engage parents (*M* = 2.88, *SD* = 0.60). Teachers’ maximum levels of satisfaction—completely satisfied—were registered regarding the interest and adequacy of the activities of the program for children. Finally, the quality of each session was also self-reported by facilitators. Items included in this dimension focused on structural quality indicators such as: the adequacy of the materials provided; the physical condition of the session space; the adequacy of the session duration; and process quality indicators such as the quality of facilitator-students relationships and quality of peer relations during each session. Each item was coded in a five-point scale, with values closer to 5 indicating a more positive quality. Overall, the facilitator reported a quality of structural aspects of the intervention on a 4.48 average level (*SD* = 0.25), and an average value of process implementation quality of 4.32 (*SD* = 0.27).

### 2.5. Data analyses

Data were analyzed using IBM SPSS version 28. Student’s *t*-test for independent samples was used to compare the mean values obtained by the groups in the pre-test and the post-test. Effect sizes were estimated and interpreted using Cohen’s *d* (*d* > 0.2 small effect, *d* > 0.5 moderate effect and *d* > 0.8 large effect, Cohen, 1988). Repeated measures analyses of covariance (ANCOVAs) were used to explore interactions between pre-post gains and group conditions. All dependent variables were successively introduced in the repeated measures factor (Within—Subject Factor), with two levels (pre- and post-test), while the group variable (experimental vs. comparison) was introduced in the independent factor (Between—Subject Factor), and child gender, as well as the mothers’ and fathers’ level of education were introduced as covariates. Effect sizes were estimated and interpreted using partial eta squared (*η^2^* > 0.01 small effect, *η^2^* > 0.06 moderate effect and *η^2^* > 0.14 large effect, Cohen, 1988).

## 3. Results

Average values obtained by the IG and the CG in the pre-test were compared using the Student’s *t* test for independent samples for all variables in the study. The results revealed the absence of statistically significant differences in all variables related to classroom peer relationships for the overall sample and separately for third and fourth graders; and in almost all variables related to SES included in the study, except for communication (reported by teachers) both for the overall sample and for fourth-graders, with CG presenting significantly higher levels of communication when compared with the IG, at pre-test, *t*(208) = −1.576, *p* < 0.001 and *t*(115) = −1.139 *p* = 0.002, respectively; and for sociability (reported by teachers) for third-graders, *t*(91) = −1,599, *p* = 0.001, also with the CG group presenting significantly higher levels of sociability according to teachers´ report.

Considering the absence of statistically significant differences between the IG and the CG at the pre-test for almost all variables, main effects of time and interactive effects of time with the group condition were examined and are presented below for variables related to SES (e.g., communication and self-regulation and its subdimensions) and variables classroom peer relationship variables (e.g., conflict and cooperation).

### 3.1. Effects of the intervention on children’s self-regulation and communication

Gain differences in all the SES are summarized in Table 1 for the total group of participants, and separately for third- and fourth-grade students in Tables 2, 3, respectively. For the overall participants, comparisons between the groups were computed for post-test data to examine main effects of group. Main effects of time were examined by analyzing intragroup growth (Table 1). Over time, there was a statistically significant increase in children’s emotional control and children sociability, reported by teachers, for the IG. For the CG, there was a significant decrease, over time, of assertiveness (reported by parents) and communication (reported by parents); and an increase in sociability, reported by teachers. This last result on sociability seems to be similar both for IG and CG. Interactive effects of time with group condition were examined. No main or interactive effects were found for self-regulation, self-control, emotional control, communication, sociability, and cooperation, as reported by students, families, and teachers. Only for assertiveness, as reported by families, an interactive effect of time and group condition was found, *F*(1,180) = 3.747, *p* = 0.05, *η^2^* = 0.020, with the IG showing greater gains in this dimension, compared to the CG. This indicates a positive effect of the intervention in students’ assertiveness. Nevertheless, a small effect size was found.

**Table 1:** Means, standard deviations, and *t*-test for paired samples, repeated measures ANOVA and magnitude of effect for socioemotional skills: whole sample.

	Intervention group				Comparison group					
	Pre-test	Post-test			Pre-test	Post-test				
Child version	M(SD)	M(SD)	*t*	*d*	M(SD)	M(SD)	*t*	*d*	*F*	*η^2^*
Self-regulation	4.47(0.67)	3.45(0.65)	0.294	0.60	3.63(0.69)	3.60(0.71)	0.455	0.65	0.219	0.001
Emotional control	3.21(0.77)	3.25(0.68)	−0.708	0.70	3.36(0.74)	3.40(0.77)	−0.497	0.67	0.067	—
Self-control	3.73(0.73)	3.67(0.77)	1.105	0.72	3.92(0.78)	3.80(0.79)	1.080	0.83	0.277	0.001
Communication	3.53(0.41)	3.50(0.49)	0.687	0.47	3.61(0.48)	3.55 (0.43)	0.879	0.48	0.161	0.001
Assertiveness	2.33(0.87)	2.40(0.93)	−1.007	0.80	2.32(0.97)	2.25(0.96)	0.600	0.96	0.646	0.003
Cooperation	4.15(0.62)	4.14(0.63)	0.180	0.65	4.33(0.72)	4.29(0.62)	0.388	0.78	0.223	0.001
Sociability	4.11(0.55)	3.97(0.67)	2.335^*^	0.70	4.18(0.68)	4.13(0.69)	0.485	0.65	0.562	0.003
Family version										
Self-regulation	3.34(0.66)	3.33(0.61)	0.342	0.46	3.38(0.65)	3.41(0.60)	−0.440	0.50	0.450	0.002
Emotional control	3.29(0.78)	3.26(0.72)	0.720	0.54	3.31(0.71)	3.35(0.68)	−0.475	0.53	0.686	0.004
Self-control	3.93(0.67)	3.40 (0.63)	−0.196	0.49	3.46(0.70)	3.49(0.65)	−0.365	0.57	0.137	0.001
Communication	3.75(0.41)	3.75(0.42)	0.187	0.32	3.86(0.40)	3.79(0.38)	1.683^+^	0.29	1.307	—
Assertiveness	3.03(0.79)	3.08(0.82)	−0.759	0.72	3.07(0.83)	2.90(0.84)	2.034^*^	0.60	3.747^*^	0.020
Cooperation	4.24(0.43)	4.20(0.41)	1.305	0.33	4.34(0.43)	4.30(0.43)	0.723	0.36	0.033	—
Sociability	3.98(0.61)	3.96(0.55)	0.488	0.41	4.16(0.56)	4.17(0.54)	−0.207	0.37	0.299	0.002
Teacher version										
Self-regulation	3.71(0.88)	3.75(0.83)	−0.872	0.59	3.63(0.87)	3.65(0.91)	−0.354	0.44	0.160	0.001
Emotional control	2.56(0.44)	2.69(0.43)	−2.133^**^	0.49	2.78(0.54)	2.81(0.46)	−0.569	0.44	2.054	0.010
Self-control	3.64(0.96)	3.73(0.86)	−1.626	0.70	3.49(0.89)	3.58(0.95)	−1.299	0.57	0.080	—
Communication	3.47(0.61)	3.47(0.52)	−0.174	0.39	3.60(0.41)	3.58(0.46)	0.793	0.25	0.418	0.002
Assertiveness	2.53(1.20)	2.51(1.32)	0.466	0.66	2.59(1.02)	2.45(1.06)	1.615	0.70	1.213	0.006
Cooperation	3.92(0.74)	3.88(0.69)	0.740	0.62	4.03(0.66)	4.01(0.72)	0.353	0.47	0.002	—
Sociability	3.94(0.72)	4.04(0.61)	−2.199^*^	0.55	4.20(064)	4.29(0.67)	−1.845^+^	0.41	0.015	—

^*^p < 0.05; ^**^p < 0.01; ^***^p < 0.001; ^+^*p* < 0.09. ^a^Child gender, maternal education, and paternal education were entered as covariates.

**Table 2:** Means, standard deviations, and *t*-test for paired samples, repeated measures ANOVA and magnitude of effect for socioemotional skills: third graders (*n* = 91).

	Intervention group				Comparison group					
	Pre-test	Post-test			Pre-test	Post-test				
Child version	M(SD)	M (SD)	*t*	*d*	M(SD)	M (SD)	*t*	*d*	*F*	*η^2^*
Self-regulation	3.54(0.68)	3.44(0.74)	1.134	0.66	3.71(0.81)	3.77(0.72)	−0.344	0.84	0.129	0.002
Emotional control	3.76(0.78)	3.64(0.83)	1.170	0.82	3.99(0.88)	4.04(0.79)	−0.205	1.03	0.080	0.001
Self-control	3.30(0.74)	3.25(0.79)	0.626	0.73	3.43(0.85)	3.50(0.77)	−0.475	0.85	0.103	0.001
Communication	3.54(0.43)	3.53(0.57)	0.132	0.51	3.63(0.52)	3.66(0.38)	−0.287	0.57	0.229	0.003
Assertiveness	2.25(1.01)	2.60(1.07)	−3.043^*^	0.90	2.54(1.06)	2.42(1.02)	0.630	1.03	6.448^**^	0.074
Cooperation	4.20(0.67)	4.10(0.70)	1.061	0.75	4.25(0.83)	4.36(0.69)	−0.702	0.93	0.391	0.005
Sociability	4.16(0.49)	3.89(0.74)	2.658^*^	0.79	4.13(0.74)	4.20(0.64)	−0.528	0.74	2.178	0.032
Family version										
Self-regulation	3.38(0.53)	3.36(0.57)	0.274	0.42	3.46(0.62)	3.39(0.70)	0.850	0.43	0.461	0.006
Emotional control	3.45(0.55)	3.42(0.59)	0.443	0.47	3.58(0.72)	3.51(0.66)	0.804	0.47	0.219	0.003
Self-control	3.30(0.66)	3.30(0.66)	−0.031	0.56	3.34(0.69)	3.27(0.86)	0.644	0.51	0.480	0.007
Communication	3.68(0.44)	3.76(0.48)	−1.594	0.36	3.89(0.42)	3.83(0.41)	1.009	0.26	3.120^+^	0.041
Assertiveness	2.99(0.82)	3.12(0.88)	−1.375	0.75	3.00(0.93)	2.86(0.90)	1.220	0.53	2.860^+^	0.032
Cooperation	4.17(0.44)	4.17(0.44)	−0.023	0.36	4.48(0.41)	4.39(0.47)	1.471	0.30	5.853^*^	0.074
Sociability	3.89(0.63)	3.98(0.59)	−1.655^+^	0.44	4.17(0.53)	4.25(0.49)	−1.145	0.31	0.213	0.003
Teacher version										
Self-regulation	3.64(0.91)	3.70(0.90)	−1.863^+^	0.64	3.40(0.73)	3.45(0.77)	−0.578	0.51	1.279	0.015
Emotional control	3.57(0.91)	3.76(0.91)	−2.079^*^	0.70	3.32(0.75)	3.51(0.75)	−1.730^+^	0.60	0.525	0.006
Self-control	2.61(0.44)	2.62(0.38)	−0.141	0.45	2.92(0.60)	3.00(0.41)	−0.815	0.52	0.350	0.004
Communication	3.41(0.53)	3.43(0.36)	−0.374	0.43	3.55(0.38)	3.49(0.43)	1.136	0.28	1.471	0.018
Assertiveness	2.34(1.13)	2.21(1.06)	1.492	0.69	2.73(1.08)	2.64(1.01)	0.557	0.82	0.211	0.003
Cooperation	3.96(0.73)	3.96(0.72)	0.000	0.52	3.84(0.70)	3.81(0.65)	0.350	0.52	0.917	0.011
Sociability	3.87(0.80)	4.11(0.62)	−2.880^*^	0.66	4.10(0.51)	4.05(0.58)	0.599	0.46	6.132^**^	0.070

^*^p < 0.05; ^**^p < 0.01; ^***^p < 0.001; ^+^*p* < 0.09. ^a^Child gender, maternal education, and paternal education were entered as covariates.

**Table 3:** Means, standard deviations, and *t*-test for paired samples, repeated measures ANOVA and magnitude of effect for socioemotional skills: fourth graders (*n* = 133).

	Intervention group				Comparison group					
	Pre-test	Post-test			Pre-test	Post-test				
Child version	M(SD)	M(SD)	*t*	*d*	M(SD)	M(SD)	*t*	*d*	*F*	*η^2^*
Self-regulation	3.42(0.67)	3.47(0.59)	−0.775	0.55	3.57(0.57)	3.46(0.68)	1.571	0.43	3.878^*^	0.036
Emotional control	3.71(0.70)	3.69(0.73)	0.344	0.64	3.85(0.69)	3.60(0.74)	2.461^*^	0.58	1.860	0.017
Self-control	3.14(0.78)	3.25(0.58)	−1.600	0.66	3.30(0.63)	3.31(0.76)	−0.167	0.48	3.575^+^	0.033
Communication	3.52(0.40)	3.48(0.42)	0.841	0.44	3.59(0.46)	3.47(0.46)	1.935^+^	0.38	0.933	0.009
Assertiveness	2.39(0.75)	2.24(0.77)	2.053^*^	0.65	2.13(0.87)	2.10(0.90)	0.199	0.91	1.195	0.011
Cooperation	4.11(0.58)	4.17(0.57)	−0.952	0.57	4.40(0.60)	4.22(0.55)	1.726^+^	0.59	4.892^*^	0.045
Sociability	4.07(0.59)	4.03(0.61)	0.559	0.62	4.22(0.63)	4.09(0.74)	1.438	0.56	1.044	0.010
Family version										
Self-regulation	3.31(0.75)	3.30(0.65)	0.219	0.49	3.34(0.67)	3.44(0.53)	−1.085	0.54	1.488	0.014
Emotional control	3.35(0.74)	3.38(0.66)	−0.602	0.52	3.38(0.69)	3.48(0.65)	−0.926	0.62	2.574	0.025
Self-control	3.28(0.86)	3.21(0.76)	1.008	0.53	3.29(0.74)	3.39(0.53)	−1.096	0.55	0.442	0.004
Communication	3.81(0.38)	3.74(0.37)	2.229^*^	0.26	3.84(0.39)	3.77(0.37)	1.332	0.31	0.072	0.001
Assertiveness	3.06(0.77)	3.04(0.78)	0.263	0.69	3.12(0.79)	2.93(0.81)	1.612	0.65	0.032	---
Cooperation	4.29(0.41)	4.23(0.39)	1.933^*^	0.30	4.25(0.42)	4.25(0.39)	−0.069	0.40	1.200	0.012
Sociability	4.05(0.59)	3.95(0.53)	2.427^*^	0.37	4.15(0.58)	4.12(0.58)	0.476	0.40	0.452	0.004
Teacher version										
Self-regulation	3.76(0.87)	3.73(0.79)	0.656	0.54	3.83(0.94)	3.82(1.00)	0.145	0.39	0.214	0.002
Emotional control	3.68(1.00)	3.71(0.82)	−0.366	0.70	3.64(0.98)	3.65(1.10)	−0.103	0.55	5.386^*^	0.047
Self-control	2.52(0.44)	2.75(0.46)	−3.931^*^	0.51	2.67(0.46)	2.66(0.45)	0.162	0.35	0.000	---
Communication	3.51(0.67)	3.51(0.62)	0.128	0.37	3.65(0.44)	3.66(0.48)	−0.070	0.23	0.036	---
Assertiveness	2.67(1.24)	2.72(1.14)	−0.760	0.63	2.48(0.98)	2.29(1.09)	1.892^+^	0.60	3.534^+^	0.031
Cooperation	3.89(0.76)	3.82(0.67)	0.883	0.69	4.20(0.58)	4.19(0.75)	0.133	0.42	0.262	0.002
Sociability	3.99(0.66)	3.99(0.61)	0.086	0.43	4.29(0.74)	4.51(0.69)	−3.894^*^	0.33	7.827^**^	0.067

^*^p < 0.05; ^**^p < 0.01; ^***^p < 0.001; ^+^*p* < 0.06. ^a^Child gender, maternal education, and paternal education were entered as covariates.

Data were examined separately for third- and fourth-grade students. For third-grade students (Table 3), a statistically significant interactive effect of time with group condition was found, supporting the hypothesis that the IG group would benefit from the intervention in terms of assertiveness *F*(1,81) = 6.448, *p* = 0.01, *η^2^* = 0.074 (child report,); and sociability, *F*(1,81) = 5.60, *p* = 0.02, *η^2^* = 0.07 (teacher report). Effects sizes were small to moderate. No effects were found on self-regulation, self-control, and emotional control, for either of the informants’ reports. For fourth-grade students (Table 4), a positive interactive effect of time and the group condition was found on self-regulation, *F*(1,105) = 3.878, *p* = 0.05, *η^2^* = 0.036 (child report); and emotional control, *F*(1,109) = 5.836, *p* = 0.02, *η^2^* = 0.047 (teacher report); Effects favoring the CG were found on sociability, *F*(1,109) = 7.827, *p* = 0.006, *η^2^* = 0.067 (teacher report). This provides partial support of the positive effect of the intervention for fourth-grade students SES, although effects sizes were small to moderate.

**Table 4:** Means, standard deviations, and *t*-test for paired samples, repeated measures ANCOVA and magnitude of effect for classroom peer relationships variables for the whole sample (*N* = 185).

	Intervention group				Comparison group					
	Pre-test	Pos-test			Pre-test	Pos-test				
	*M(SD)*	*M(SD)*	*t*	*d*	*M(SD)*	*M(SD)*	*t*	*d*	*F* *^a^*	*η2*
Comfort	4.56(0.47)	4.37(0.76)	2.850^**^	0.77	4.57(0.61)	4.58(0.53)	−0.168	0.62	1.040	0.005
Cooperation	3.73(0.92)	3.87(0.93)	−1.514	1.01	3.94(0.95)	3.79(0.79)	1.425	0.85	4.643^*^	0.024
Conflict	2.99(1.07)	2.82(0.94)	1.825^+^	1.10	2.91(1.04)	3.09(0.90)	−1.504	0.98	5.045	0.026
Mutual affection	2.51(1.27)	2.33(1.24)	1.226	1.60	2.26(1.19)	2.72(1.09)	−2.683^**^	1.34	7.103^*^	0.037
Cohesion	3.76(0.93)	3.70(0.93)	0.658	1.02	3.81(1.04)	3.67(0.86)	1.376	0.91	0.911	0.05
Isolation	2.34(0.96)	2.44(0.88)	−1.085	1.06	2.38(0.91)	2.61(0.86)	−1.949^+^	0.93	0.753	0.004

^*^p < 0.05; ^**^p < 0.01; ^***^p < 0.001.

a Child gender, maternal education, and paternal education were entered as covariates.

### 3.2. Effects on classrooms peer relationships

Building on the absence of statistically significant differences between the IG and the CG at the pre-test, comparisons between the groups were computed for post-test data to examine main effects of group. Overall, results showed statistically significant differences between the groups at the post-test for levels of comfort, conflicts, mutual affection, and isolation. More specifically, the CG showed higher levels of comfort and mutual affection at the post-test, when compared to the IG, *t*(204) = −1.98, *p* = 0.049 and *t*(199) = −2.07, *p* = 0.039, respectively. The IG presented lower levels of conflicts and isolation at post-test, when compared to the CG, *t*(204) = −2.12, *p* = 0.032 and *t*(199) = −2.07, *p* = 0.039, respectively.

Moreover, the main effects of time were examined (Table 4). For the IG, over time, there was a statistically significant decrease in children’s perception of comfort in the classroom, *t*(139) = −2.850, *p* = 0.005, *d* = 0.77. For the CG a statistically significant increase in level of mutual affection, *t*(63) = −2.683, *p* = 0.009, *d* = 1.34, from pre- to post-test. Furthermore, to understand if changes at post-test were due to the participation in the SEL intervention, six individual repeated measures ANCOVAs were conducted, exploring interactive effects of time with condition (IG or CG) for the overall group pf participants. A statistically significant interactive effect of time with group condition, supporting the hypothesis that the IG would present a more positive change in the variables, emerged for the following dimensions: level of conflicts, *F*(1, 191) = 5.045, *p* = 0.02, *η^2^* = 0.026, and cooperation, *F*(1, 191) = 4.643, *p* = 0.003, *η^2^* = 0.024. A statistically significant interactive effect of time with group condition, favoring the CG, was found on the levels of mutual affection, *F*(1, 202) = 7.103, *p* = 0.0008, *η^2^ = 0*.037. No effects were found for the dimensions of comfort, isolation, and cohesion (Table 4).

Data were examined separately for third- and fourth-grade students. No effects were found on the variable regarding classroom peer relations (Table 5) for third-grade students. For fourth-grade students (Table 6), a statistically significant interactive effect of time and the group condition was found on conflict, *F*(1, 105) = 7.013, *p = 0*.009, *η^2^* = 0.063, with a significant advantage for the IG that sees levels of conflicts decreasing over time, while CG sees levels of conflict increasing. Also, an interactive effect, favoring the IG, is documented on levels of isolation, with CG having an increase of isolation levels, while the IG maintain the initial isolation levels, *F*(1, 105) = 5.248, *p* = 0.024, *η^2^* = 0.048; in cooperation, *F*(1, 105) = 9.886, *p* = 0.002, *η^2^* = 0.086, with the IG showing significant gains, while the CG decreases level of cooperation over time; and in levels of cohesion, *F*(1, 105) = 4.286, *p =* 0.016, *η^2^* = 0.037, with the IG increasing their levels of cohesion, while the CG decreases in this variable (Table 6). An interactive effect of time and group condition, favoring the CG, was encountered on mutual affection. No effects were found on levels of comfort (Table 6).

**Table 5:** Means, standard deviations, and *t*-test for paired samples, repeated measures ANCOVA and magnitude of effect for classroom peer relationships variables for third graders (*n* = 91).

	Intervention group				Comparison group					
	Pre-test	Pos-test			Pre-test	Pos-test				
	*M(SD)*	*M(SD)*	*t*	*d*	*M(SD)*	*M(SD)*	*t*	*d*	*F* *^a^*	*η^2^*
Comfort	4.45(0.54)	4.20(0.79)	2.280^*^	0.86	4.41(0.62)	4.20(0.79)	−1.90^+^	0.66	7.451^**^	0.084
Cooperation	3.63(0.92)	3.66(1.01)	−0.231	0.92	3.59(0.97)	3.66(1.01)	−0.46	0.86	0.004	---
Conflict	3.21(1.04)	3.18(0.93)	0.181	1.06	3.21(1.03)	3.19(0.93)	−0.11	1.07	0.011	0.003
Mutual affection	2.77(1.33)	2.72(1.24)	0.245	1.58	2.80(1.35)	2.75(1.26)	−1.93^+^	1.64	3.705^+^	0.044
Cohesion	3.69(0.98)	3.45(0.93)	1.99^*^	0.93	3.65(1.02)	3.45(0.93)	−0.30	1.92	1.049	0.013
Isolation	2.47(1.02)	2.70(0.92)	−1.780^+^	1.03	2.48(1.01)	2.71(0.92)	0.25	0.97	0.962	0.012

^*^p < 0.05; ^**^p < 0.01; ^***^p < 0.001; ^+^*p* < 0.06.

a Child gender, maternal education, and paternal education were entered as covariates.

**Table 6:** Means. standard deviations, and *t*-test for paired samples.

	Intervention group				comparison group					
	Pre-test	Pos-test			Pre-test	Pos-test				
	*M(SD)*	*M(SD)*	*t*	*d*	*M(SD)*	*M(SD)*	*t*	*d*	*F* *^a^*	*η^2^*
Comfort	4.65(0.40)	4.51(0.71)	1.728	0.70	4.73(0.39)	4.55(0.44)	1.977^+^	0.52	1.294	0.012
Cooperation	3.81(0.91)	4.02(0.82)	−1.722	1.08	4.21(0.71)	3.86(0.66)	2.549^*^	0.80	9.886^**^	0.086
Conflict	2.82(1.07)	2.53(0.85)	2.225^*^	1.13	2.68(0.98)	3.00(0.65)	−2.143^*^	0.89	7.013^**^	0.063
Mutual affection	2.30(1.19)	2.04(1.17)	1.402	1.64	2.12(1.07)	2.47(0.87)	−1.938^+^	1.00	4.095^*^	0.039
Cohesion	3.81(0.88)	3.89(0.88)	−0.665	1.08	4.09(0.61)	3.75(0.73)	2.637^*^	0.76	6.039^*^	0.054
Isolation	2.24(0.89)	2.23(0.80)	0.079	1.07	2.15(0.86)	2.63(0.84)	−3.269^*^	0.83	5.248^*^	0.048

Repeated measures ANCOVA and magnitude of effect for classroom peer relationships for fourth graders (*n* = 113). ^*^p < 0.05; ^**^p < 0.01; ^***^p < 0.001; ^+^*p* < 0.06.

a Child gender, maternal education, and paternal education were entered as covariates.

## 4. Discussion

This study used a quasi-experimental design to analyze the effects of a universal SEL intervention, delivered as part of the school curriculum, on elementary school students’ self-regulation, communication and classroom peer relationships, within a multi-informant approach. Although results revealed some inconsistency across informants and dimensions, some support for positive effects of the intervention on students´ competencies is provided. Overall, students who participated in this SEL program improved in dimensions of SES, such as self-regulation and communication, as well as in dimensions of classroom peer relationships, such as peer conflicts and peer cooperation, when compared with children who did not participate. For the overall sample, assertiveness, as reported by families, emerged as the competence in which students participating in the intervention showed more gains, compared to students not participating in the intervention. Moreover, based on children’s reports, there was a positive effect of the program on classroom peer conflicts and cooperation in the classroom, with students participating in the program reporting a significant decrease in levels of conflicts in classroom, and higher levels of cooperation, when compared to students who did not participate in the intervention. As mentioned, results from this study are mixed, with positive effects of the intervention found for some dimensions, but not consistently across informants and dimensions. The same pattern, i.e., mixed results are also reported in the literature regarding the effects of universal SEL intervention programs on students, with small to moderate effect sizes being described (Carroll et al., 2020; Merrin and Low, 2023). For instance, and similarly to our study, Raimundo and Pinto (2016) found, in their quasi-experimental exploratory study with elementary school students, significant gains in SES, including peer relations and social competence. Nevertheless, other studies (e.g., Kim et al., 2015) found no effects of intervention in elementary school students’ engagement behaviors after a SEL intervention. The literature documents the potential of SEL universal interventions to support all students of a given school or grade to enhance intra and interpersonal competences (e.g., Greenberg et al., 2003), albeit some students identified at risk or with social and emotional problems could benefit from additional targeted support. Moreover, the diversity of students based on personal and contextual characteristics can influence the participation and benefit of universal intervention (Cipriano et al., 2023). Thus, it is expected heterogeneous results due to students do not benefit equally from universal interventions (Merrin and Low, 2023). This can reinforce the need for continuous systems of support to students, which universal intervention can be complemented by delivering targeted interventions that fit students’ specific needs (Cipriano et al., 2023).

Additionally, for the group of students participating in the intervention, our results also show a decrease for some of the outcomes the intervention aimed to improve. Although this was not expected according to our hypotheses. One possible explanation for this may be related to fact that after a SEL intervention students report lower levels of SES competences which can be due to gains in the awareness of what are SES and what are the indicators of positive SES. By improving children emotional literacy and self-awareness, students may get more demand both regarding their own SES as well as regarding the assessment of their classroom peer relationships quality. Thus, it would be important that future studies could further explore these explanations, by using a qualitative approach to understand student’s experiences during SEL interventions, as well as individual meanings and criteria during self-assessments.

Recent literature underlines the need of research to consider the study of differential gains for children participating in SEL interventions, exploring how these programs affect the development of different groups of children. Most studies are exploring subgroups based on the participants socio-demographic characteristics (e.g., gender and socioeconomic level) which can be considered narrow (Simmons et al., 2018) based on the complexity of schools settings and in context developmental processes (Osher et al., 2020). In this scope, and having in consideration that children in third and fourth grades face different academic and socioemotional challenges, the effects of the program were analyzed separately for third and fourth grade students. Academically, third-grade students are usually in a stage where their literacy competencies are being consolidated, with communication and social interaction skills associated with the development of language and literacy competences being simultaneously challenged and potentiated. As for the fourth-grade students, this is the last year of elementary school and so the emotional challenges associated with the transition to the next level of education (fifth grade) is underlined in this period. The last year of elementary school is key for the development of regulatory skills—both emotional and behavior regulation. Thus, the effectiveness of the intervention program was examined separately for third and fourth grade students. A statistically significant positive effect of the program was found on third-grade students’ assertiveness (child report), and sociability, (teacher report), as well as on the following classroom peer relations dimensions: comfort in classroom and mutual affection. For fourth-grade students, findings showed a statistically significant positive effect of the intervention on self-regulation (child report), emotional control (teacher report), as well as on the following classroom peer relations dimension: level of conflicts in classroom and levels of mutual affection.

While the positive indicators of the SEL intervention program show potential for improving children’s competencies and peer contexts in the classroom, there remains inconsistency among informants in reporting the effectiveness of the program. Therefore, additional research is necessary, particularly with regard to the implementation process, in order to better understand the potential of this specific intervention. It is important to identify the factors that may have contributed to the inconsistent results across informants, such as differences in perception or understanding of the intervention or the influence of other contextual factors. Further investigation about the implementation process can shed light on these factors, which in turn can inform the development of more effective interventions. Several authors have identified the difficulties in demonstrating SEL intervention results, arguing that the effectiveness of the SEL programs is closely linked with the quality of the intervention process (e.g., Durlak et al., 2011; Bierman and Motamedi, 2015; Taylor et al., 2017; Voith et al., 2020; Mahoney et al., 2021; Ștefan et al., 2022; Wigelsworth et al., 2022). In our study, we underline that several monitoring mechanisms were implemented with data showing positive indicators of fidelity and responsiveness, although the intervention process may be affected by the pandemic. Even though the intervention delivered in this study did not follow all recommended guidelines from international literature, i.e., the intervention was only delivered for one school year not including the entire school calendar and had to be adapted for online during the pandemic (Durlak et al., 2011), the program analyzed in this study seems to be promising, with some positive effects on several relevant students’ competencies. Recognizing that SEL is a developmental and individually based process, it is expected that certain competences may be acquired easily while others may require more support and instruction (Ura et al., 2020), resulting in variance in competences level. Therefore, we hypothesize that the results of this study may have been affected by the intervention intensity, i.e., to improve some skills a more continuous intervention over time might be needed. Moreover, we recognize that the program implemented included a wide set of socioemotional skills and was not exclusively focused on self-regulation, communication and peer classroom relationships, which may also have affected its ability to produce changes in the outcome variables. Additionally, note that although four of the 16 intervention sessions were adapted to the online format, both regarding the strategies and goals for the sessions, these adaptations were consistent across intervention groups and closely supervised by the author of the program, adjusting to the real-life needs. Despite the adjustment of the program to the pandemic may have affected the effectiveness of the intervention as several studies underline that SEL programs need to be implemented effectively, with high-quality, evidence-based instructions in order to improve children’s SES and development (e.g., Durlak et al., 2011; Sklad et al., 2012; Kim et al., 2015; Wigelsworth et al., 2022), we also underline that this was crucial to adequately respond to schools, students and family’s needs in a crises period. Nevertheless, future studies are needed to examine the potential positive effects of “Calmly - Learning to Learn Yourself” [Calmamente—Aprendendo a Aprender-se] SEL program on students’ competences when delivered across all school year, in a full face-to-face format (as it was originally designed) and using larger samples, increasing both the number of classrooms and schools in each condition, and including different school systems (e.g., private and public, suburban and rural).

Research has also been stressing that SEL programs that are embedded in the school environment as a whole are more effective in promoting children’s competencies, rather than just having curriculum based SEL interventions (e.g., Wigelsworth et al., 2022). For instance, Adi et al. (2007) found evidence favoring whole-school, multicomponent intervention programs, underlining the positive effects of such interventions when compared to solely curriculum based SEL intervention programs. The authors found that teacher training and professional development, as well as parenting support during SEL interventions had a particularly differential positive effect on children’s mental health outcomes. Despite mixed evidence regarding the differential effectiveness of interventions based on their action level (e.g., Wigelsworth et al., 2022), with several limitations regarding the own definition and ability of the studies to capture the whole school processes, we underline that, in the present study, the intervention was infused into the third and fourth-grade students’ curricula but implemented by an external professional. Even though teachers and parental involvement was preconized in the intervention rationale, these were not consistently planned and consistently supported through the intervention in order to enhance an integrated approach of SEL across key settings of students. As such, a true whole school embedded intervention was not delivered in this study and we hypothesize that this may have affected the intervention efficacy, along with the above mentioned constrains during intervention. Although the intervention delivered in this study is among the few SEL interventions, in Portugal, that are infused into the curricula, we also note that a long path is still to come for in-depth changes in the school environments and curricula organization to align with the CASEL recommendations for SEL universal interventions and daily practices in schools.

### 4.1. Study limitations

This study provides preliminary evidence for the potential effectiveness of the “Calmly - Learning to Learn Yourself” [Calmamente—Aprendendo a Aprender-se] SEL program, however results must be interpreted carefully and some limitations must be acknowledged. Therefore, caution must be exercised when interpreting the results, and they should not be overgeneralized to other contexts, with the need of further research to confirm and expand our findings, as well as to explore the effectiveness of the program with different populations and in different settings. Given the heterogeneity of intervention outcomes, future research should consider a person-centered approach for identifying personal and contextual variables related to program effectiveness and for tracking different patterns of changes. The number of participants was limited with an uneven number of children in IG and CG. Future studies are needed with larger samples and balanced groups in terms of the number of participants. Then the implementation of the program only lasted 16 sessions/weeks over the course of one school year; the intervention did not start at the beginning of the school year, had to be adapted to the online format during the COVID-19 pandemic, and was only delivered in one school cluster. Additionally, although the program rationale considered the need to foster the generalization of the competencies promoted through teacher’s extension of the program’s activities, no control over how teachers implemented this aspect of the program was documented. This lack of control is problematic as it may have resulted in inconsistent implementation of the strategies in daily activities. To address this issue, future iterations of the program could include specific guidance and training for teachers on how to promote the use of the strategies and how to monitor implementation of the program in daily activities. Future studies should monitor how teachers embedded the program strategies in their classes and in their interaction with children in order to analyze differential effects of the program based on such extension. Furthermore, results from self-report measures completed by children may be affected by their reading and comprehension levels. For instance, third graders showed some difficulties in reading some questionnaire items. Results from teachers’ reports may also be interpreted carefully as both the IG and the CG school were part of the same school cluster and social desirability may have affected their responses. Although teacher ratings are generally considered as a valid method for documenting children’s competencies, it is possible that teachers’ knowledge of the experimental condition may have influenced results. It is recommended that future studies not only use a multiformat approach, as the one used on the present study, but also include observation measures of students in their natural environments to capture the effects of the interventions (Cooke et al., 2007). Including observational assessments could also contribute to overcome the measurement issues related to SES. Additionally, the measure used to assess students’ SES—the SSES—is not validated for Portuguese children. Although showing good reliability, future studies are needed on this measure for Portuguese samples. Moreover, literature underlines that there is no standardized approach to measuring social and emotional skills (Merrell, 2010; Durlak et al., 2011), and so we must recognize that our measures may not captured such skills accurately, hindering the efforts to capture short term results of the interventions (Ura et al., 2020).

### 4.2. Conclusion

Theoretical and empirical evidence supports the assumption that SEL universal interventions are crucial in educational settings. Despite a growing interest in understanding and supporting SEL in schools, including in Portugal, the definition and scope of SEL interventions remain broad, with mixed evidence across studies. As the emphasis on the universal SEL approaches where all students and adults in schools are engaged in a coordinated learning process (Durlak et al., 2022) is reinforced, the need for more evidence on the effects of infused SEL interventions into the school curricula also grows. The present study focused on a recently developed Portuguese SEL intervention for elementary school children, providing initial evidence of its impact on children’s competencies and classroom climate-related variables. To our knowledge, this is among the first quasi-experimental studies conducted in Portugal to analyze the effects of a SEL program infused into the curriculum for elementary school-aged children. Although several challenges in the development of a coherent set of evidence were faced, and further research about the intervention features and implementation is required, the initial results show that the intervention contributes to some of children’s socio-emotional competencies and school peer classroom climate variables. However, the inconsistency of the present study results needs to be acknowledged. The unexpected pandemic that emerged while the intervention was being delivered posed additional challenges to both the program’s original design (face-to-face intervention) implementation and the study features, potentially affecting the intervention’s effectiveness. Therefore, in-depth changes in school environments and curricula organization are still required to align with CASEL recommendations for SEL universal interventions and daily practices in schools.

## Data availability statement

The raw data supporting the conclusions of this article will be made available by the authors, without undue reservation.

## Ethics statement

Ethical review and approval was not required for the study on human participants in accordance with the local legislation and institutional requirements. Written informed consent to participate in this study was provided by the participants’ legal guardian/next of kin.

## Author contributions

VC and CP contributed equally to the study design and manuscript. CP, VC, and FM made substantial contributions to the conception or design of the study, analysis, and interpretation of data. AE led the intervention team and is responsible for the conception of the intervention program. VC, HA, CP, MS, and FM made substantial contributions in drafting the paper and revising it critically. VC, CP, HA, FM, MS, and AE were involved in ensuring that questions related to the accuracy or integrity of any part of the work were appropriately investigated and resolved. All authors contributed to the article and approved the submitted version.

## Funding

This work was supported by the Calouste Gulbenkian Foundation, particularly within the scope of the “Gulbenkian Academies for Knowledge” [Academias do Conhecimento] program and also by the Portuguese Foundation for Science and Technology (UIDB/00050/2020).

## Conflict of interest

The authors declare that the research was conducted in the absence of any commercial or financial relationships that could be construed as a potential conflict of interest.

## Publisher’s note

All claims expressed in this article are solely those of the authors and do not necessarily represent those of their affiliated organizations, or those of the publisher, the editors and the reviewers. Any product that may be evaluated in this article, or claim that may be made by its manufacturer, is not guaranteed or endorsed by the publisher.

## Footnotes

^1^ https://gulbenkian.pt/academias/
